
JOURNAL INFORMATION
==============================
Journal ID: Wirtschaftsdienst

Wirtschaftsdienst

EISSN: 1613-978X

ARTICLE INFORMATION
==============================
PMCID: PMC8933168
DOI: 10.1007/s10273-022-3127-2
Article ID: 3127
Article version: 1
Subjects: Zeitgespräch

Nach Corona kommt die Schuldenwelle — begründete Sorge oder Panikmache? Aktuelle Entwicklungen im Kredit- und Konsummarkt

After Corona Comes the Debt Wave — Well-Founded Concern or Scaremongering?

Donau Kai-Friedrich kai-friedrich.donau@schufa.de

Teamleiter CSR, SCHUFA Holding AG, Kormoranweg 5, 65201 Wiesbaden, Deutschland
Electronic publication date: 2022 Mar 19
Print publication date: 2022
Volume: 102
Issue: 3
First page: 166
Last page: 169
Copyright: © Der/die Autor:in 2022
License: Open Access: Dieser Artikel wird unter der Creative Commons Namensnennung 4.0 International Lizenz veröffentlicht (https://creativecommons.org/licenses/by/4.0/deed.de).
Open Access wird durch die ZBW — Leibniz-Informationszentrum Wirtschaft gefördert.
License URL: https://creativecommons.org/licenses/by/4.0/

Keywords: E21, G51, G53
issue-copyright-statement: © The Author(s) 2022

==============================

Die Coronapandemie hat tiefe Spuren bei den Verbraucher:innen hinterlassen. Die schwierige Situation in zahlreichen Wirtschaftsbranchen hat zu finanziellen Belastungen vieler Haushalte geführt. Entgegen wiederholter Warnungen vor einer Schuldenwelle lagen die neuen Zahlungsstörungen in den vergangenen zwei Jahren jedoch gemäß der Auswertung der SCHUFA-Daten unterhalb des Niveaus der Vor-Corona-Zeit. Der Grund: Die Verbraucher:innen haben ihr Finanz- und Konsumverhalten angepasst oder haben auf ihre Rücklagen zurückgegriffen – Rücklagen, die nun allerdings angesichts steigender Preise vor allem bei der Energie fehlen könnten.

Als im Frühjahr 2020 ein neues Virus Deutschland erreichte und sich rasant verbreitete, war die Hoffnung noch groß, durch verschiedene Maßnahmen die Ausbreitung der Krankheit unter Kontrolle zu bekommen und die Auswirkungen auf Gesellschaft und Wirtschaft gering zu halten. Ein Trugschluss: Die Pandemieentwicklung führte zu mehreren Lockdowns und zahlreichen Einschränkungen des Alltagslebens. Über die wirtschaftlichen und sozialen Folgen dieser Maßnahmen konnte damals aufgrund der sich ständig verändernden und extrem dynamischen Entwicklung nur gemutmaßt werden. Besondere mediale und politische Aufmerksamkeit erhielt die finanzielle Situation der Verbraucher:innen: Die Coronakrise traf große Teile der Wirtschaft und vor allem den Arbeitsmarkt hart. Viele Menschen mussten in Kurzarbeit gehen oder verloren ihren Job – etwa in der Tourismuswirtschaft, dem Kulturbereich oder der Gastronomie. Vor diesem Hintergrund wurde von verschiedenen Seiten vor einem Schulden-Tsunami gewarnt, einer Welle an Privatinsolvenzen. Hat sich dies als begründete Sorge herausgestellt oder war es Panikmache?

## Analysen zum Konsum- und Kreditverhalten

Ein möglicher Weg, sich der Antwort dieser Frage zu nähern, ist die Auswertung der konsum- und kreditbezogenen Daten der vergangenen zwei Pandemiejahre. Der Datenbestand der SCHUFA umfasst zahlungsrelevante Informationen, beispielsweise über Leasingverträge, Ratenzahlungsgeschäfte oder Kredite zu 68 Mio. natürlichen Personen und 6 Mio. Unternehmen. Auf dieser Basis wertet die SCHUFA einmal jährlich den gesamten Datenbestand des Vorjahrs aus, um das Konsum- und Kreditverhalten der deutschen Verbraucher:innen zu analysieren.

Darüber hinaus werden seit 2020 auch monatlich einige zentrale Kennzahlen zum Kreditverhalten in der Coronakrise abgebildet, die ebenfalls auf Auswertungen des SCHUFA-Datenbestands basieren. Seit Beginn der Coronapandemie regelmäßig durchgeführte, repräsentative Umfragen unter Verbraucher:innen und Unternehmer:innen ergänzen diese Ergebnisse um weitere wichtige Aspekte. Auf diese Weise werden für Politik und Forschung notwendige Informationen und Stimmungsbilder bereitgestellt, um die wirtschaftlichen Auswirkungen der Coronakrise frühzeitig abschätzen zu können.

## Kreditaufnahme: Große Schwankungen 2020

Der SCHUFA Risiko- und Kredit-Kompass für das Gesamtjahr 2020 zeigt, dass die Deutschen etwa 17,6 Mio. Ratenkredite hatten, die damit eine wichtige und genutzte Finanzierungsform für größere Konsumausgaben darstellen. Die durchschnittliche Kredithöhe lag bei 12.988 Euro. Der größte Anteil der neu abgeschlossenen Ratenkredite (43,7 %) entfiel auf höhere Kreditsummen über 10.000 Euro. Die durchschnittliche Laufzeit neuer Kredite lag bei 54 Monaten. Innerhalb des Jahres 2020 gab es bei der Kreditaufnahme erhebliche Schwankungen, die im Wesentlichen von der Coronakrise beeinflusst waren. So waren etwa viele Kontaktpunkte, an denen Ratenkreditverträge geschlossen werden, wie etwa Elektromärkte, über längere Zeit geschlossen. Rückblickend ging während des ersten Lockdowns zwischen März und Juli die Nachfrage nach neuen Kreditverträgen stark zurück, hatte sich aber bis Ende 2020 erholt und das Niveau von 2019 teilweise sogar übertroffen.

Wenig überraschend profitierte der Online-Handel erheblich von der Krise: Vor dem Hintergrund eingeschränkter Einkaufsmöglichkeiten in den Geschäften verlagerten Deutschlands Verbraucher:innen ihren Konsum während der Pandemie ins Internet. Dies spiegelt sich auch in der hohen Zahl der SCHUFA-Anfragen aus dem eCommerce wider, die zeitweise 50 % höher als im Vorjahreszeitraum lag. Insgesamt setzt sich der langfristige Trend zum Online-Shopping fort und erhielt durch die Coronapandemie noch mal einen Schub.

## Zahlungsstörungen auch in der Pandemie auf niedrigem Niveau

Bei der Rückzahlung der Ratenkredite sind die deutschen Verbraucher:innen sehr zuverlässig. Besondere – negative – Einflüsse durch die Coronapandemie sind nicht zu erkennen: 2020 wurden, wie in den beiden Vorjahren, 97,9 % der Kredite ordnungsgemäß bedient. Die regionale Verteilung folgte ebenfalls längerfristigen Entwicklungen und stand in keinem Zusammenhang etwa mit Ansteckungsraten oder lokalen Pandemiemaßnahmen. So waren 2020 in den südlichen Bundesländern Bayern und Baden-Württemberg, wie in den Jahren zuvor, die wenigsten Zahlungsschwierigkeiten zu verzeichnen, während in den Bundesländern Bremen und Berlin der Anteil der Menschen mit Zahlungsschwierigkeiten am höchsten war.

Der Trend sinkender Zahlungsstörungen bei Privatpersonen setzte sich im Jahr 2021 fort (vgl. Abbildung 1). Um die Entwicklung der Zahlungsstörungen genau im Blick zu behalten und diese Erkenntnisse mit der Öffentlichkeit zu teilen, veröffentlicht die Schufa Holding AG auf ihrem Corona-Dashboard (Schufa, o.J.) regelmäßig einen Index der Personen mit neuen Zahlungsstörungen. Hier lässt sich gut erkennen, dass der Indexwert 2021 fast durchgängig unterhalb des Mittelwerts von 2019 lag.

## Insolvenzzahlen: hohe Dynamik durch Neuregelungen

Die Insolvenzzahlen der vergangenen beiden Jahre waren hingegen von einer wesentlich höheren Dynamik gekennzeichnet – allerdings nicht infolge der Coronapandemie. Bereits seit längerem ist ein Trend sinkender Verbraucherinsolvenzen zu beobachten. Mitte des Jahres 2020 sank deren Zahl rapide ab, die Privatinsolvenzen gingen insgesamt gegenüber 2019 um ein Drittel zurück (Statista, 2021).

Der starke Rückgang an Privatinsolvenzen in der Coronazeit lässt sich vor allem durch die Gesetzesreform des Insolvenzrechts erklären: Bei Privatinsolvenzen, die ab dem 1. Oktober 2020 beantragt wurden, ist schon nach drei – und nicht wie zuvor nach sechs Jahren – eine Restschuldbefreiung möglich. Es ist anzunehmen, dass viele

Abbildung 1
**Neue Zahlungsstörungen Ende 2021**
1,0=Jahresmittelwert 2019

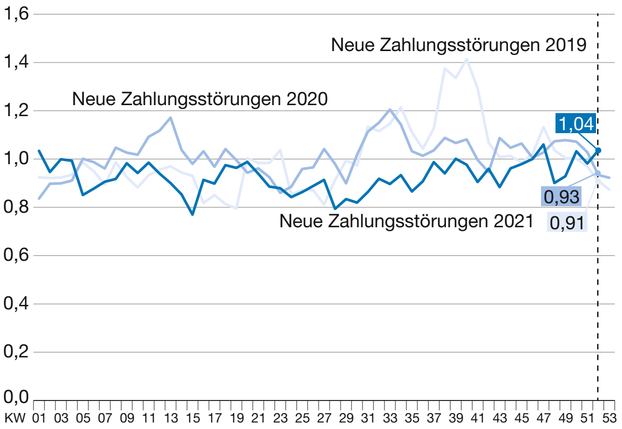

Die Zahl der Personen mit neuen Zahlungsstörungen ist gering und bewegt sich etwa auf dem Indexwert des Vor-Corona-Jahres 2019.

Quelle: SCHUFA Holding AG.

Betroffene ihren Insolvenzantrag bei Ankündigung dieser Gesetzesreform im Verlauf des Jahres 2020 hinausgeschoben haben. Darüber hinaus scheint durch die verkürzte sogenannte Wohlverhaltensphase eine Privatinsolvenz eine Option für Menschen geworden zu sein, denen der Zeitraum von sechs Jahren als zu lang erschien. Und nicht zuletzt waren viele Beratungsangebote Corona-bedingt eingeschränkt, die mit entsprechender Zeitverzögerung nachgeholt wurden. Diese Annahmen werden durch die Entwicklung im Jahresverlauf 2021 untermauert. In der ersten Jahreshälfte stiegen die Privatinsolvenzen sprunghaft an und lagen bereits höher als im gesamten Vorjahr (Statista, 2021). Allerdings schwächte sich diese Entwicklung bis Ende 2021 wieder erheblich ab.

## Einkommenseinbußen bei 30 % bis 40 % der Verbraucher:innen

Die insgesamt sehr erfreuliche Situation bei den Zahlungsstörungen bedeutet jedoch nicht, dass die Coronapandemie keine finanziellen und wirtschaftlichen Spuren bei den Verbraucher:innen hinterlassen hätte. Um dies zu erfassen, führt die Schufa seit September 2020 regelmäßig Umfragen unter Verbraucher:innen durch. Diese werden von Nordlight Research im Auftrag der Schufa durchgeführt. Nordlight Research ist ein marktpsychologisches Forschungsinstitut mit Sitz in Hilden bei Düsseldorf. Die 1.000 Teilnehmenden der Befragung werden in einem reichweitenstarken Online-Panel rekrutiert und nach Alter, Geschlecht, Haushaltsnettoeinkommen und Bundesland quotiert. Die Ergebnisse sind repräsentativ für deutschsprachige Verbraucher:innen mit Internetzugang ab 18 Jahren.

Über alle Erhebungen hinweg berichten 30 % bis 40 % der Befragten von Einbußen im Haushaltseinkommen. Diese können beispielsweise auf coronabedingte Einschränkungen der Berufstätigkeit oder auf Kurzarbeit zurückzuführen sein, welche die befragten Verbraucher:innen selbst oder andere Personen im eigenen Haushalt betreffen. Dabei geben die meisten betroffenen Verbraucher:innen an, dass sie während der Coronapandemie leichte (bis 15 %) oder mittlere (bis 30 %) Einbußen erlitten haben.

### Junge und Einkommensschwache besonders betroffen

Allerdings ist hier ein differenzierter Blick notwendig. So zeigte sich im Rahmen der Befragung vom März 2021, dass vor allem junge Menschen und einkommensschwache Haushalte besonders unter der Coronapandemie und den damit verbundenen Auswirkungen für die Bevölkerung leiden. 49 % der befragten 18- bis 25-Jährigen gaben Verluste beim Einkommen an, in der Gesamtbevölkerung liegt dieser Anteil zu diesem Zeitpunkt bei 38 %. Und 46 % der Haushalte mit einem Nettoeinkommen von unter 2.000 Euro monatlich hatten Sorge, in den folgenden sechs Monaten finanzielle Verluste zu erleiden, vor allem, falls die Lockdowns verlängert werden sollten. Unter den Haushalten mit einem Nettoeinkommen über 4.000 Euro monatlich befürchteten dies nur 31 %.

Neben den sehr konkreten Auswirkungen wie Einkommensverlusten ließen sich im Verlauf der Pandemie auch Veränderungen in der wirtschaftlichen Stimmung nachweisen. Diese war von starken Schwankungen geprägt, die vom Pandemieverlauf, den getroffenen Gegenmaßnahmen, aber auch dem Fortschreiten der Impfkampagne beeinflusst wurden. Aber auch die Stimmung war wiederum abhängig vom Haushaltseinkommen der befragten Personen.

### Anpassungsstrategien bei finanziellen Engpässen

Relativ früh in der Pandemiephase zeigte sich, dass die Verbraucher:innen auf finanzielle Engpässe durch die oben genannten Einkommensverluste reagieren und ihr Finanzverhalten in vielerlei Hinsicht an die gegebenen wirtschaftlichen Umstände anpassen: Am häufigsten wurden bereits im Vorfeld geplante, größere Anschaffungen verschoben, gleich danach folgen der Zugriff auf Rücklagen und das Überziehen des eigenen Kontos, um pandemiebedingte finanzielle Engpässe zu überbrücken (vgl. Abbildung 2).

Etwas weniger genutzt werden das Stunden von Zahlungen (außer Mieten/Ratenzahlungen für die Wohnung) und die Inanspruchnahme staatlicher Hilfen. Die Zahl der befragten Verbraucher:innen, die aufgrund der Pandemiesituation einen Kredit aufgenommen haben, ist verhältnismäßig gering. Am seltensten werden Miet- oder Ratenzahlungen für die eigene Wohnung ausgesetzt. Diese Angaben wiesen im weiteren Verlauf der Pandemie einige Schwankungen auf, dennoch blieben diese Muster weitgehend gleich. Bemerkenswert ist, dass ein Zehntel der Befragten angibt, sich Geld bei Freunden oder Verwandten geliehen zu haben. Auch hier zeigen sich je nach Haushaltsnettoeinkommen Unterschiede: Bei Einkommen von unter 2.000 Euro liegt dieser Wert bei 16 %, bei Einkommen von über 4.000 Euro nur bei 4 %.

Abbildung 2

**Finanzielle Anpassungen während der Coronakrise**

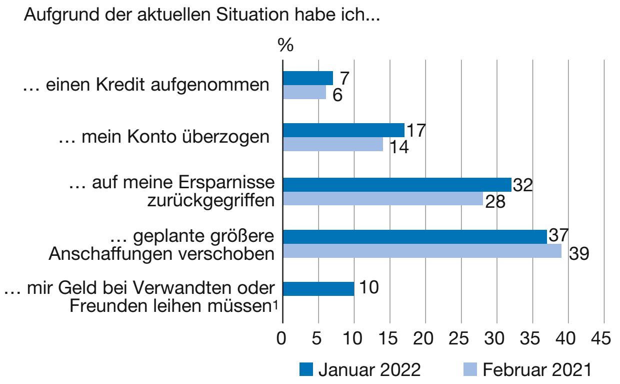

[1] Frage wurde im Februar 2022 nicht gestellt.

Quelle: SCHUFA Holding AG.

### Veränderungen im Konsum

Darüber hinaus zeigen die Umfragedaten aus dem Risiko- und Kredit-Kompass 2020, dass Einkommenseinbußen häufig zu Verzicht führen, wie sich an den Ausgaben der Verbraucher:innen ablesen lässt. So reduzierten die Befragten im ersten Pandemiejahr 2020 ihre Ausgaben am meisten in den Bereichen Reisen (67 %), Ausgehen (64 %) und kulturelle Veranstaltungen (59 %). Allerdings ist hier zu bedenken, dass Reisen und Kulturveranstaltungen auch kaum möglich waren. Für Kleidung (37 %) und sonstige Konsumgüter (23 %) gaben die Verbraucher:innen weniger aus. Jeder Fünfte spendete weniger. 17 % sparten auch im Zusammenhang mit dem eigenen Pkw. Deutlich seltener reduzierten Verbraucher:innen ihr Budget für Lebensmittel und Vereinsbeiträge (jeweils 12 %) (Schufa, 2020).

Viele Verhaltensänderungen werden auch langfristig Bestand haben. Im Januar 2022 gaben 80 % der Befragten an, dass sich ihr Einkaufsverhalten nicht „normalisiert“ hat. Leidtragende sind dabei vor allem der stationäre Handel, die Gastronomie und die Kulturszene: 48 % der Verbraucher:innen kaufen vor allem über das Internet ein, 53 % sagen, dass sie aktuell deutlich weniger auswärts essen gehen als normalerweise, und 60 % der Befragten besuchen weniger Kulturevents oder Konzerte. Die Coronapandemie hat somit das Konsumverhalten in Deutschland zum Teil grundlegend verändert.

## Fazit

Die Analysen unserer Daten und der Beobachtungs- und Befragungsdaten verdeutlichen, dass die Verbraucher:innen in Deutschland auch in schwierigen Zeiten ihre finanziellen Möglichkeiten im Blick haben und verantwortungsvoll mit Geld und Krediten umgehen. Das deutsche Kreditsystem hat unter den Coronabedingungen seine Widerstandsfähigkeit und Stabilität bewiesen. Hinzu kommen politische Maßnahmen wie das Kurzarbeitergeld oder Hilfen für Selbständige, die durchaus dazu beigetragen haben, die wirtschaftlichen Folgen der Coronakrise abzufedern. Kurz gesagt: Eine pandemiebedingte Schuldenwelle ist aktuell nicht zu beobachten. Dies ist aber kein Grund zur Entwarnung. Es gibt einzelne Gruppen von Vebraucher:innen, namentlich junge Menschen und einkommensschwache Haushalte, die von der Wirtschaftskrise massiv getroffen wurden und deren finanzielle Situation schwierig ist. Verschärfend kommt hinzu, dass ein Teil der Verbraucher:innen nur durch Rückgriff auf Ersparnisse die direkten Folgen der Pandemie abfedern konnte.

Diese Rücklagen könnten nun aber fehlen, wie unsere Erhebung aus dem Zeitraum Januar 2022 zeigt: Aktuell fürchten Menschen vor allem allgemeine Preisanstiege (74 %). Wenn es um Energiekosten geht – z. B. für Strom und Benzin – vermuten sogar 81 % der Umfrageteilnehmer:innen, dass sich die Preisspirale weiter nach oben dreht. Steigende Lebenshaltungskosten haben direkte Auswirkungen auf die persönliche Situation der Menschen: 44 % der Verbraucher:innen geben an, nicht genug Spielraum zu haben, um bei dieser Entwicklung ihren Lebensstandard weiter halten zu können. Mehr als ein Viertel der Befragten (28 %) glaubt sogar, dass es ihnen zunehmend schwerfallen wird, ihren Lebensunterhalt zu bestreiten (Schufa, 2022).

Auch wenn eine kurzfristige Schuldenwelle ausgeblieben ist, wird die Coronakrise nicht ohne längerfristige Folgen bleiben. Es gilt, speziell diese Gruppen, also Menschen mit niedrigem Haushalteinkommen und junge Menschen, künftig besonders im Auge zu behalten – und zu unterstützen.

The corona pandemic had a considerable impact on consumers. The difficult situation in numerous sectors of the economy has led to financial burdens for many households. Despite repeated warnings announcing significantly increasing debts, the number of new payment arrears during the past two years has been below the level of the pre-COVID-19 period according to analysis based on SCHUFA data. This is due to the fact that consumers have adjusted their financial and consumption behavior or made use of their financial reserves — reserves that could now be lacking due to rising prices, especially for energy.

Dr. Kai-Friedrich Donau ist Teamleiter CSR bei der SCHUFA Holding AG in Wiesbaden.


Literatur

Schufa (2020), SCHUFA Risiko- und Kredit-Kompass 2020, https://www.schufa.de/media/images/10_die_schufa/130_presse/pressemittei-lungen_2/skk_20/SCHUFA_Risiko-und-Kredit-Kompass.pdf (25. Februar 2022).
Schufa (2022), SCHUFA Corona-Update #10, https://www.schufa.de/ueber-uns/presse/pressemitteilungen/schufa-corona-update-10.jsp (25. Februar 2022).
Schufa (o.J.), SCHUFA Risiko- und Kredit-Kompass Aktuell, www.schufa-kreditkompass.de (25. Februar 2022).
Statista (2021), Privatinsolvenzen in Deutschland bis 2021, https://de.statista.com/statistik/daten/studie/150565/umfrage/privatinsol-venzen-in-deutschland-seit-2000/ (25. Februar 2022).
